# Tuberculosis-Associated Erythema Nodosum

**DOI:** 10.7759/cureus.20184

**Published:** 2021-12-05

**Authors:** Jennifer Laborada, Philip R Cohen

**Affiliations:** 1 Dermatology, University of California Riverside School of Medicine, Riverside, USA; 2 Dermatology, University of California, Davis Medical Center, Sacramento, USA

**Keywords:** tuberculosis, quantiferon, ppd, nodules, mycobacterium, infectious disease, erythema nodosum

## Abstract

Erythema nodosum is panniculitis that is frequently observed in women aged 18 to 34 years. It usually occurs as an idiopathic condition; however, it may be associated with drugs, infections, malignancy, pregnancy, and systemic illnesses. Erythema nodosum presents with the sudden onset of tender, warm, erythematous nodules typically on the ankles, knees, and shins. Although the pathogenesis has not been fully elucidated, evidence supports a delayed type IV hypersensitivity reaction. It is often a clinical diagnosis that does not require a biopsy; appropriate work-up and careful medication history are crucial to identifying an underlying etiology if present. This report describes a woman from Vietnam, a tuberculosis endemic country, who presented with erythema nodosum that was determined to be a sequela of latent tuberculosis. Several studies have demonstrated an association between erythema nodosum and tuberculosis, especially in endemic regions. Summarized data reveals the incidence of tuberculosis-associated erythema nodosum to be six percent; however, when individuals with either secondary erythema nodosum or infection-associated erythema nodosum are evaluated, the incidence of tuberculosis-associated erythema nodosum is 11% or 21%, respectively. Evaluation of erythema nodosum should include a tuberculin or QuantiFERON test, chest roentgenogram, and/or an acid-fast bacilli sputum culture if the diagnosis of tuberculosis is being considered.

## Introduction

Erythema nodosum is septal panniculitis characterized by erythematous nodules on the lower extremities. It can occur as an idiopathic skin disease. However, it is often the cutaneous stigmata of a systemic condition or drug-adverse effect [1-13].

Tuberculosis is a mycobacterial infection that is endemic in certain areas of the world, such as Southeast Asia, India, and South Africa. It clinically presents with chronic cough, sputum production, chest pain, or shortness of breath. Often, systemic symptoms, such as fever, night sweats, weight loss, and fatigue, are present [14,15].

We describe a woman who presented with erythema nodosum. Her workup discovered latent tuberculosis. Tuberculosis-associated erythema nodosum is reviewed.

## Case presentation

A 59-year-old Vietnamese woman presented with a six-week history of red and painful swollen nodules on the distal legs; she denied other symptoms of cough, dysuria, fatigue, fever, malaise, sore throat, and weight loss. Her primary care physician suspected cellulitis and prescribed a 10-day course of doxycycline 100 mg twice per day. He also referred her to be evaluated by a dermatologist.

Her past medical history is significant for attention-deficit/hyperactivity disorder, herpes zoster, hypertension, migraines, obesity, and seborrheic dermatitis. She never received the Bacillus Calmette-Guérin (BCG) vaccine, and she lives with her husband and daughter. She has been on clonidine and amlodipine for hypertension for several months, and no new medications before the onset of her skin lesions had been initiated. A colonoscopy performed a year ago only observed normal findings.

**Figure 1:**
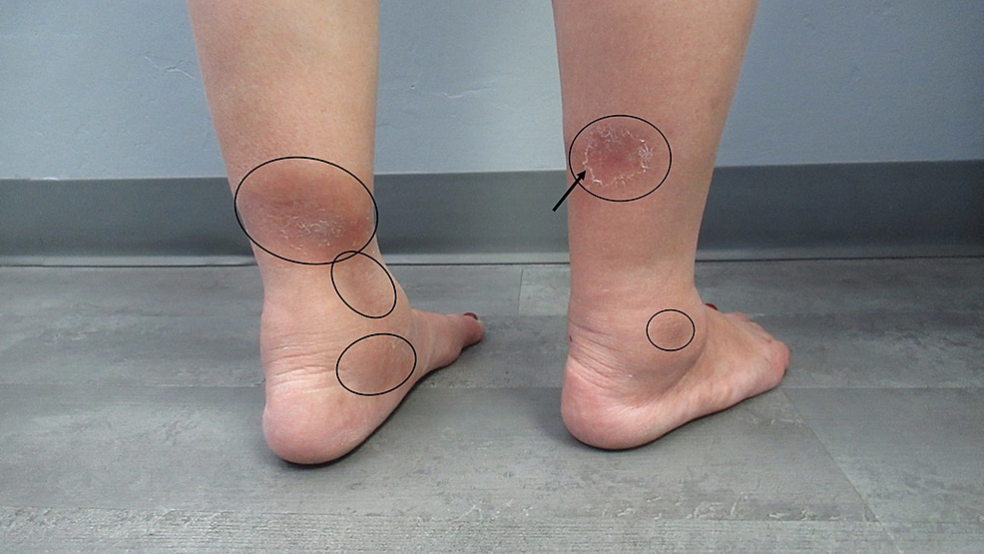
Erythema nodosum associated with latent tuberculosis Hyperpigmented, slightly raised nodules (black ovals) with focal desquamation (black arrow) of six weeks duration on the posterior and lateral distal legs of a 59-year-old Vietnamese woman with latent tuberculosis.

**Figure 2:**
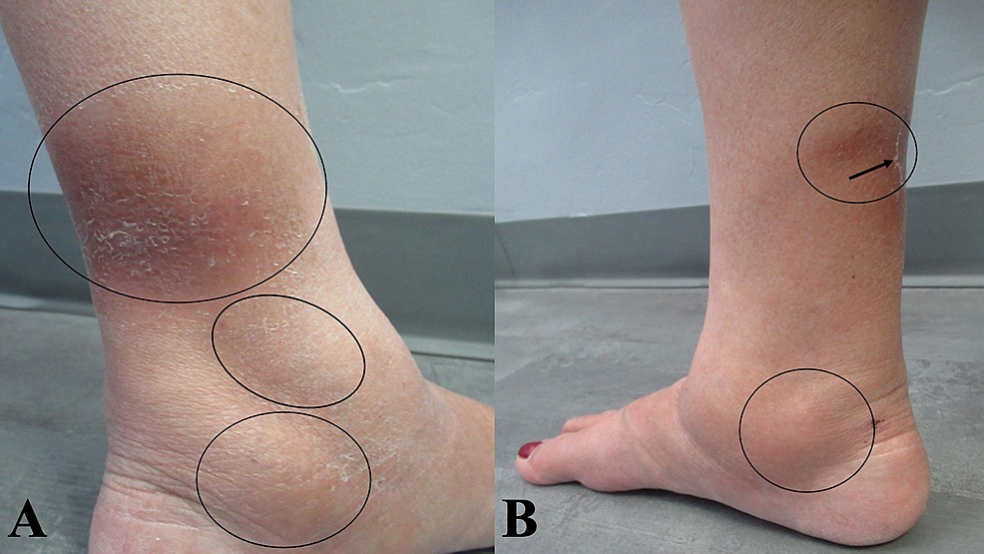
Tuberculosis-associated erythema nodosum A closer view of three tender nodules (black ovals) on the medial left distal leg (A) and two painful nodules (black ovals) with focal desquamation (black arrow) on the medial right distal leg (B).

Cutaneous examination revealed tender hyperpigmented nodules on the bilateral distal lower extremities near the ankles. There was desquamation of the skin overlying some of the areas of resolving redness (Figures 1, 2). Based on the clinical presentation, a diagnosis of erythema nodosum was established.

Laboratory evaluation for potential erythema nodosum-associated conditions was performed. The following laboratory studies were either normal or negative: complete blood count, chemistry serum panel, erythrocyte sedimentation rate, antistreptolysin O, human immunodeficiency virus, hepatitis B surface antigen, hepatitis C antibody, and stool culture (for campylobacter, salmonella, Shiga toxin, and shigella). However, her C-reactive protein was elevated at 16.2 mg/L (reference: < 8.0 mg/L). A tuberculosis skin test (PPD) test was not performed; her QuantiFERON gold was positive. Although her chest roentgenogram revealed a mild basilar infiltrate of the right middle lobe, her sputum culture was negative for *Mycobacterium tuberculosis*. 

Correlation of the clinical presentation with laboratory studies suggested a diagnosis of tuberculosis-associated erythema nodosum. She was evaluated by an infectious disease specialist who confirmed the diagnosis of latent tuberculosis; her abnormal chest roentgenogram was not considered to be related to tuberculosis. Daily treatment was initiated with isoniazid 300 mg and pyridoxine 50 mg for a planned duration of six months. At follow-up two months later, there were no new erythema nodosum lesions. All of her lesions had flattened with residual post-inflammatory hyperpigmentation. 

## Discussion

Erythema nodosum is considered type IV delayed hypersensitivity response to various antigens. Even though its pathogenesis is not fully understood, evidence suggests that the mechanism involves immune complex deposition in the septal venules of the subcutaneous fat, neutrophil recruitment with the production of reactive oxygen intermediates, and tumor necrosis factor (TNF), and granuloma formation. Erythema nodosum typically presents on distal lower extremities as painful tender nodules; however, in some individuals, the lesions are located more diffusely. Similar to our patient, it can be misdiagnosed as cellulitis and treated-often unsuccessfully-with oral antibiotics [1,12].

The diagnosis of erythema nodosum is often made clinically. A biopsy can be performed to confirm septal panniculitis and exclude other possibilities. If a skin biopsy is performed for microscopic evaluation, consideration for sending additional specimens that have not been placed in formalin for tissue culture (such as bacterial, fungal, mycobacterial) should be entertained [1,11].

Once the diagnosis of erythema nodosum is established, additional evaluation to determine if there is an associated etiology should be considered. Currently, there is no standard workup for patients with erythema nodosum. Similar to our patient, it is reasonable to do the following tests: beta-human chorionic gonadotropin blood test (in women), complete blood count, comprehensive metabolic serum panel, erythrocyte sedimentation rate, thyroid function tests (thyroid-stimulating hormone [TSH], thyroxine [T4], and triiodothyronine [T3]), stool culture (for bacteria), and a tuberculosis skin test (PPD) or QuantiFERON. Infectious etiologies should also be ruled out by testing for human immunodeficiency virus, hepatitis B antigen, hepatitis C antibody, and antistreptolysin O; a chest roentgenogram may be considered to evaluate for lymphoma, sarcoidosis, and tuberculosis. In addition, a colonoscopy may be performed to evaluate for inflammatory bowel diseases such as Crohn’s disease and ulcerative colitis [1-13].

The etiology of erythema nodosum is diverse. From retrospective studies, idiopathic erythema nodosum ranges from nine percent to 72% (median, 34%) (Table 1) [2-12]. Secondary erythema nodosum can be associated with drugs, infections (bacterial, fungal, mycobacterial, parasitic, and viral), malignancy (hematologic and solid tumors), pregnancy, and systemic diseases such as inflammatory bowel disease and sarcoidosis [1-13]. Differential diagnoses for tuberculosis-associated erythema nodosum include the following: alpha-1 antitrypsin deficiency, cutaneous polyarteritis nodosa, erythema induratum of Bazin, erythema nodosum leprosum, lupus panniculitis, panniculitis-like T cell lymphoma, superficial thrombophlebitis, and pancreatic panniculitis [1].

**Table 1:** Incidence of tuberculosis-associated erythema nodosum EN: erythema nodosum; Inf: infection-related erythema nodosum; pts: patients; Ref: reference; Tot: total; TB: tuberculosis; 1°: primary; 2°: secondary; #: number ^a ^This is the percent of erythema nodosum patients who had tuberculosis-associated erythema nodosum. ^b ^This is the percent of secondary erythema nodosum patients whose erythema nodosum was related to tuberculosis. ^c ^This is the percent of infection-associated erythema nodosum patients in whom the infection was tuberculosis.

Author	Year	# TB pts	# Tot pts	% Tot pts^a^	# 1° EN pts	# 2° EN pts	% 2° EN^b^ pts	# Inf pts	% Inf pts^c^	Ref
Erez	1987	1	44	2	14	30	3	20	5	[2]
Puavilai	1995	12	100	12	72	28	43	18	67	[3]
Cribier	1998	1	129	1	71	58	2	42	2	[4]
Garcia-Porrua	1999	5	102	5	35	67	7	34	15	[5]
Psychos	2000	2	132	2	46	86	2	12	17	[6]
Sota	2004	10	45	22	15	30	33	26	38	[7]
Mert	2004	5	50	10	23	23	22	15	33	[8]
Mert	2007	10	100	10	53	47	21	21	48	[9]
Varas	2015	6	91	7	29	62	10	44	14	[10]
Babamahmoudi	2016	0	21	0	4	17	0	8	0	[11]
Porges	2019	0	45	0	4	41	0	10	0	[12]
Total		52	859	6	366	489	11	250	21	

Tuberculosis is caused by *Mycobacterium tuberculosis*. A review of retrospective erythema nodosum studies reveals that tuberculosis-associated erythema nodosum ranges from zero percent to 22% (median, five percent). When the data is combined, six percent (52 of 859 patients) of erythema nodosum was associated with tuberculosis (Table 1) [2-12].

The incidence of tuberculosis increases when specific groups of erythema nodosum patients are evaluated. Tuberculosis was 11% (52 of 489 patients) of secondary erythema nodosum. When patients with infection-associated erythema nodosum are considered, tuberculosis was 21% (52 of 250 patients) of these individuals (Table 1) [2-12].

There are two clinical subtypes of tuberculosis: active disease and latent infection. Latent tuberculosis infection exists when individuals are infected with *Mycobacterium tuberculosis* but are asymptomatic, non-infectious, and without any radiological abnormality or microbiological evidence of disease. Latent tuberculosis infection comprises more than 90% of persons infected with *Mycobacterium tuberculosis* and requires appropriate treatment. If untreated, around five to 15 percent of infected persons will develop active disease, especially those with a weakened immune system [14].

The active disease occurs when the tuberculosis bacteria overcome the immune system and begin multiplying in the body. Those with active disease are infectious and symptomatic. Key symptoms include chest pain, chills, chronic cough, fatigue, fever, hemoptysis, night sweats, and weight loss. Individuals may also have abnormal chest radiography or a positive sputum smear or culture [14].

There is no gold standard diagnostic test for latent tuberculosis, but it can be diagnosed indirectly with either a tuberculin skin test or an interferon-gamma release assay, which examines the cellular immune response to mycobacterial antigens. Given these two tests are unable to distinguish between latent and active tuberculosis, the gold standard for diagnosis of active tuberculosis is a sputum culture. Supplementary diagnostic tools include nucleic acid amplification testing, imaging, and histopathological examination of biopsy samples [15].

Our patient presented with erythema nodosum. The diagnostic evaluation was significant for an elevated C-reactive protein and a positive QuantiFERON. Her chest roentgenogram showed a mild right middle lobe infiltrate; it was not considered to be tuberculosis-related since her sputum culture was negative. She was referred to an infectious disease specialist who confirmed the diagnosis of latent tuberculosis, and treatment with anti-mycobacterial therapy was initiated. She has not had any new or recurrent erythema nodosum lesions.

## Conclusions

Erythema nodosum is panniculitis that presents as painful lesions on the distal extremities. Although its presentation can mimic a soft tissue infection, the diagnosis can often be established clinically. Erythema nodosum may be idiopathic; however, associated etiologies for erythema nodosum can be evaluated by performing a complete history and laboratory evaluation. Our patient’s workup discovered latent tuberculosis. The incidence of tuberculosis-associated erythema nodosum, from a review of several retrospective erythema nodosum studies, is six percent; however, the incidence of tuberculosis is higher when only individuals with secondary erythema nodosum (11%) or infection-associated erythema nodosum (21%) are considered. Once the diagnosis of tuberculosis is established, appropriate antimycobacterial treatment should be initiated.
